# Causal relationship between lactate dehydrogenase and risk of developing ischemic stroke: A Mendelian randomized study

**DOI:** 10.1002/brb3.3352

**Published:** 2023-12-31

**Authors:** Fuxiang Dong, Xu Wang, Jinjian Li, Dexi Zhao, Jinhua Li

**Affiliations:** ^1^ College of Traditional Chinese Medicine Changchun University of Chinese Medicine Changchun Jilin China; ^2^ School of Public Health Jilin University Changchun Jilin China

**Keywords:** ischemic stroke, lactate dehydrogenase, Mendelian randomization

## Abstract

**Background and objective:**

Ischemic stroke (IS) is one of the major global health problems. It is not clear whether there is a causal relationship between lactate dehydrogenase (LDH) and the risk of IS attacks. The purpose of this study was to investigate whether LDH has a causal relationship with the development of IS.

**Methods:**

The genome‐wide association data of LDH and IS were obtained through a Mendelian randomization‐based platform. Single nucleotide polymorphisms (SNP) that were significantly associated with LDH were identified and used as instrumental variables, and a two‐sample Mendelian randomization study was used to examine the causal relationship between LDH and IS. The statistical methods included Inverse‐variance weighted approach, MR‐Egger regression, and weighted median estimator.

**Results:**

We selected 15 SNPs of genome‐wide significance from Genome‐wide association study database with LDH as instrumental variables. A consistent causal association between LDH and IS was observed by different assessment methods. The results of the inverse‐variance weighted method suggested an inverse association between LDH and higher genetic predictability of IS risk (OR, 0.997; 95%CI 0.995–0.999). The weighted median estimate showed consistent results with the MR‐Egger method (weighted median estimate: OR, 0.995; 95%CI 0.992–0.999; MR‐Egger method: OR, 0.996; 95%CI 0.992–0.999). The inverse‐variance weighted method indicates a causal association between LDH and IS (β = −0.002563, SE = 0.00128, *p* = .0453). MR‐Egger analysis (β = −0.004498, SE = 0.001877, *p* = .03) and the weighted median method suggested that LDH and IS also existed causal relationship (β = −0.004861, SE = 0.001801, *p* = .00695).

**Conclusions:**

Our Mendelian randomization results suggest that LDH is inversely associated with the risk of developing IS, and are contrary to the results of previous observational studies.

## Graphical abstract

Our Mendelian randomization results suggest that LDH is negatively associated with the risk of developing IS, and are contrary to the results of previous observational studies.

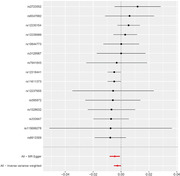

## 1 INTRODUCTION

Stroke is a common disease and one of the leading causes of death worldwide. Currently, about 80 million people worldwide suffer from stroke, and the stroke burden is the second highest in the world (Feigin et al., 2022). Strokes can be classified as hemorrhagic strokes and ischemic strokes (IS), with IS accounting for more than 80% of all strokes (Moretti et al., 2015).

Lactate dehydrogenase (LDH) is an enzyme that catalyzes the reduction and oxidation reaction between pyruvate and lactate, accompanied by the interconversion of NADH and NAD(+) (Wiwanitkit, 2007). Although LDH is normally found in low concentrations in the blood, LDH is produced in all tissues, so there are multiple reasons for elevated LDH levels (Livesey et al., 2020). It has been found that IS is followed by elevated LDH. However, the relationship between LDH and poor IS outcomes remains unclear. In the current studies, some studies suggested that high LDH was associated with poor IS outcomes (Jin et al., 2022), including hemorrhagic transformation (Chen et al., 2023) and cognitive impairment (Xu et al., 2022). Conversely, it has also been suggested that LDH was not associated with adverse IS outcomes (Sharma et al., 2021). Furthermore, the causal relationship between LDH and IS remains largely unclear. And assessing the causal relationship between LDH and IS will be very challenging due to the presence of confounding factors or reverse causality bias in observational studies.

Mendelian randomization (MR) is a method that uses genetic variation as an instrumental variable for exposure factors. It is a method used to infer a causal relationship between exposure factors and outcomes. This method is widely used to assess the causal effect of exposures on clinical outcomes (Ference et al., 2019). Since genetic variants follow Mendelian laws and are randomly distributed in the population, the effects of confounding factors are largely controlled. Thus, the MR method overcomes some limitations of traditional epidemiological studies (Hammer et al., 2009). Therefore, we performed a two‐sample MR analysis to explore the causal relationship between elevated LDH and IS. We selected single nucleotide polymorphisms (SNPs) associated with LDH levels as instrumental variables (IVs).

## 2 MATERIALS AND METHODS

### 2.1 Data sources for genetic variants of LDH and IS

Public data on LDH‐related gene variants were obtained from MR Base database (app.mrbase.org/), which contains extensive summary statistics of genome‐wide association studies (GWAS). We used the publicly available summarized statistics data sets of GWAS for LDH in individuals (*n* = 126,329; ID: bbj‐a‐30) as the exposure. The *p*‐value threshold for the two‐sample MR study of genetic variants associated with LDH in this study was 5.00E‐08, and the LD Rsq threshold was 0.001. We obtained summarized statistics including β coefficients and standard errors (SE) for 15 SNPs associated with LDH as the IVs. These SNPs include rs12316441, rs11611373, rs12226999, rs2723552, rs6547692, rs10844773, rs3129987, rs7941845, rs115699278, rs12237655, rs595872, rs1528632, rs333947, rs6913309, and rs12230154. We took a GWAS publicly available summarized statistics set for IS excluding all hemorrhages (*n* = 3314, ID: ukb‐d‐I9_STR_EXH).

### 2.2 MR analysis of LDH and IS

MR analysis needs that genetic variants were correlated with exposure rather than with potential confounders (Burgess et al., 2013). First, we evaluated the independent correlations between SNPs and LDH, SNPs and IS risk. Further, we used MR analysis to estimate the causal relationship between LDH and IS. We performed a two‐sample MR, one that used summary statistics from different GWAS to rate the causal effect of exposure (LDH) on outcome (IS) (Hartwig et al., 2016). We used pooled GWAS data from LDH and IS with 15 SNPs as IVs to assess the causal relationship between LDH and IS risk. The inverse variance weighted (IVW) approach uses meta‐analysis methods to combine causal effects from different SNPs as well as to provide consistent estimates of the causal effect of exposure on outcome when each genetic variant meets the IV hypothesis (Pierce & Burgess, 2013). Estimates of the causal relationship between LDH and IS were expressed as odds ratio (OR) with its 95% confidence interval (CI), and *p* < .05 was considered statistically significant. All MR analyses used in this study were done on the MR Base platform (App version:1.4.3 8a77eb [October 25, 2020]), R version:4.0.3) (Hemani et al., 2018).

### 2.3 Sensitivity analysis

We examined the sensitivity of the results using the leave‐one‐out method (i.e., removing individual SNPs one by one and calculating the effect of the remaining SNPs by the IVW method). In this way, we investigated the effect of individual SNPs on causal inference (Mikshowsky et al., 2017). Further, we used weighted median and MR‐Egger regression methods to explore and correct for pleiotropy.

## 3 RESULTS

### 3.1 Detail Information of the Included SNPs

Detailed information of each SNP is shown in Table 1, including effect allele (EA) and effect allele frequency (EAF). The association estimates of each SNP with LDH and IS including β‐values, SE and *p*‐values are also listed in Table 1. Fifteen of the SNPs, namely rs12316441 (β = 0.001; SE = 0.0006; *p* = .04), rs11611373 (β = 0.001; SE = 0.0006; *p* = .04), rs12226999 (β = 0.0003; SE = 0.0004; *p* = .49), rs2723552 (β = −0.0003;SE = 0.0002; *p* = .16), rs6547692 (β = 0.0001; SE = 0.0002; *p* = .49), rs10844773 (β = −7.82E‐06; SE = 0.0003; *p* = .98), rs3129987 (β = −2.31E‐06; SE = 0.0003; *p* = .99), rs7941845 (β = 8.66E‐05; SE = 0.0003; *p* = .78), rs115699278 (β = −0.0004; SE = 0.001; *p* = .74), rs12237655 (β = 0.0002; SE = 0.0006; *p* = .70), rs595872 (β = −0.0002; SE = 0.0003; *p* = .56), rs1528632 (β = −0.0002; SE = 0.0002; *p* = .42), rs333947 (β = 0.0003; SE = 0.0003; *p* = .27), rs6913309 (β = −0.0003; SE = 0.0003; *p* = .18), rs12230154 (β = 0.0002; SE = 0.0002; *p* = .18), significantly correlated with LDH. A threshold value of F < 10 is used to define “weak IV.” Therefore, the weak instrumental bias in the results is negligible.

**TABLE 1:** Characteristics of the SNPs Associated with LDH and associations with IS.

SNPs	EA	EAF	LDH			IS		
			β	SE	*p*	β	SE	*p*
rs12316441	T	0.16	−0.25	0.005	1.00E‐200	0.001	0.0006	.04
rs11611373	A	0.1575	−0.25	0.005	1.00E‐200	0.001	0.0006	.04
rs12226999	C	0.243	0.11	0.004	6.62E‐122	0.0003	0.0004	.49
rs2723552	G	0.701	−0.02	0.005	4.89E‐09	−0.0003	0.0002	.16
rs6547692	A	0.4387	0.03	0.004	3.86E‐10	0.0001	0.0002	.49
rs10844773	C	0.183	−0.05	0.005	1.47E‐24	−7.82E‐06	0.0003	.98
rs3129987	C	0.8848	−0.03	0.006	2.00E‐08	−2.31E‐06	0.0003	.99
rs7941845	T	0.4159	−0.03	0.004	2.95E‐10	8.66E‐05	0.0003	.78
rs115699278	T	0.0634	0.05	0.008	6.12E‐09	−0.0004	0.001	.74
rs12237655	A	0.2132	−0.04	0.005	1.38E‐14	0.0002	0.0006	.70
rs595872	A	0.7783	0.027	0.005	3.60E‐08	−0.0002	0.0003	.56
rs1528632	G	0.2815	0.026	0.004	4.63E‐09	−0.0002	0.0002	.42
rs333947	A	0.2947	−0.05	0.004	2.90E‐23	0.0003	0.0003	.27
rs6913309	A	0.1314	0.04	0.006	9.17E‐14	−0.0003	0.0003	.18
rs12230154	C	0.7811	0.05	0.005	1.29E‐29	0.0002	0.0002	.18

### 3.2 MR results

**FIGURE 1:**
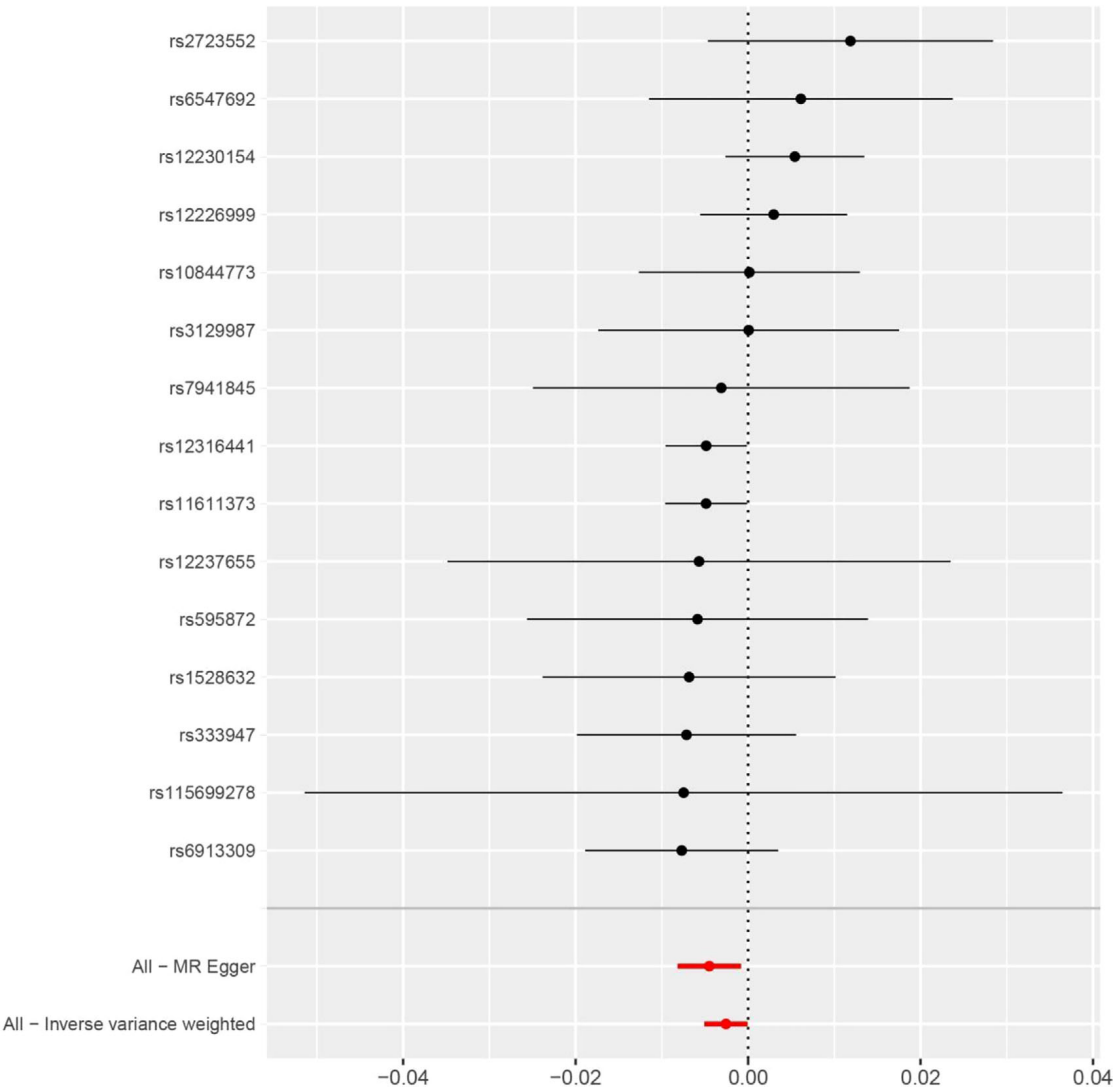
Forest plot of SNPs associated with LDH and the risk of IS, including rs12316441, rs11611373, rs12226999, rs2723552, rs6547692, rs10844773, rs3129987, rs7941845, rs115699278, rs12237655, rs595872, rs1528632, rs333947, rs6913309, and rs12230154.

**FIGURE 2:**
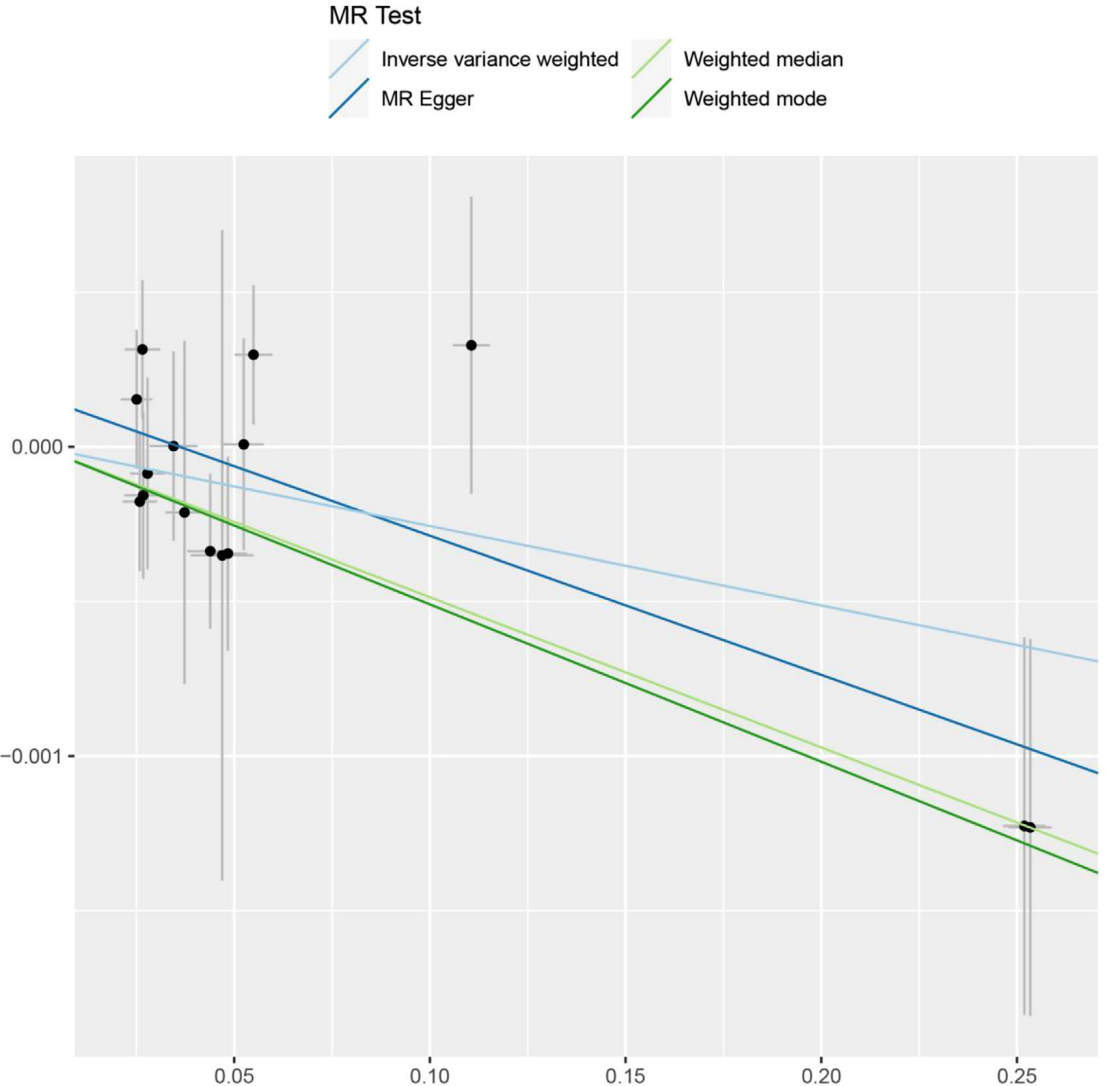
Scatter plots of genetic associations with LDH against the genetic associations with IS.

The IVW method suggested a causal association between LDH and IS (β = −0.002563, SE = 0.00128, *p* = .0453). MR‐Egger analysis (β = −0.004498, SE = 0.001877, *p* = .03) and the weighted median method showed that LDH and IS also existed causal relationship (β = −0.004861, SE = 0.001801, *p* = .00695). As shown in Table 2, the results of the IVW method suggested an inverse association between LDH and higher genetic predictability of IS risk (OR = 0.997, 95% CI = 0.995–0.999). The weighted median estimate showed consistent results with the MR‐Egger method (weighted median estimate: OR = 0.995, 95% CI = 0.992–0.999; MR‐Egger method: OR = 0.996, 95% CI = 0.992–0.999). These results are also shown in the forest plot (Figure 1) and scatter plot (Figure 2).

**TABLE 2:** Causal associations between genetically determined LDH and IS.

MR method	β	SE	OR (95% CI)	*p*
MR Egger	−0.004498	0.001877	0.996 (0.992–0.999)	.03231
Weighted median	−0.004861	0.001801	0.995 (0.992–0.999)	.00695
Inverse variance weighted	−0.002563	0.00128	0.997 (0.995–0.999)	.0453

### 3.3 Sensitivity analysis

**FIGURE 3:**
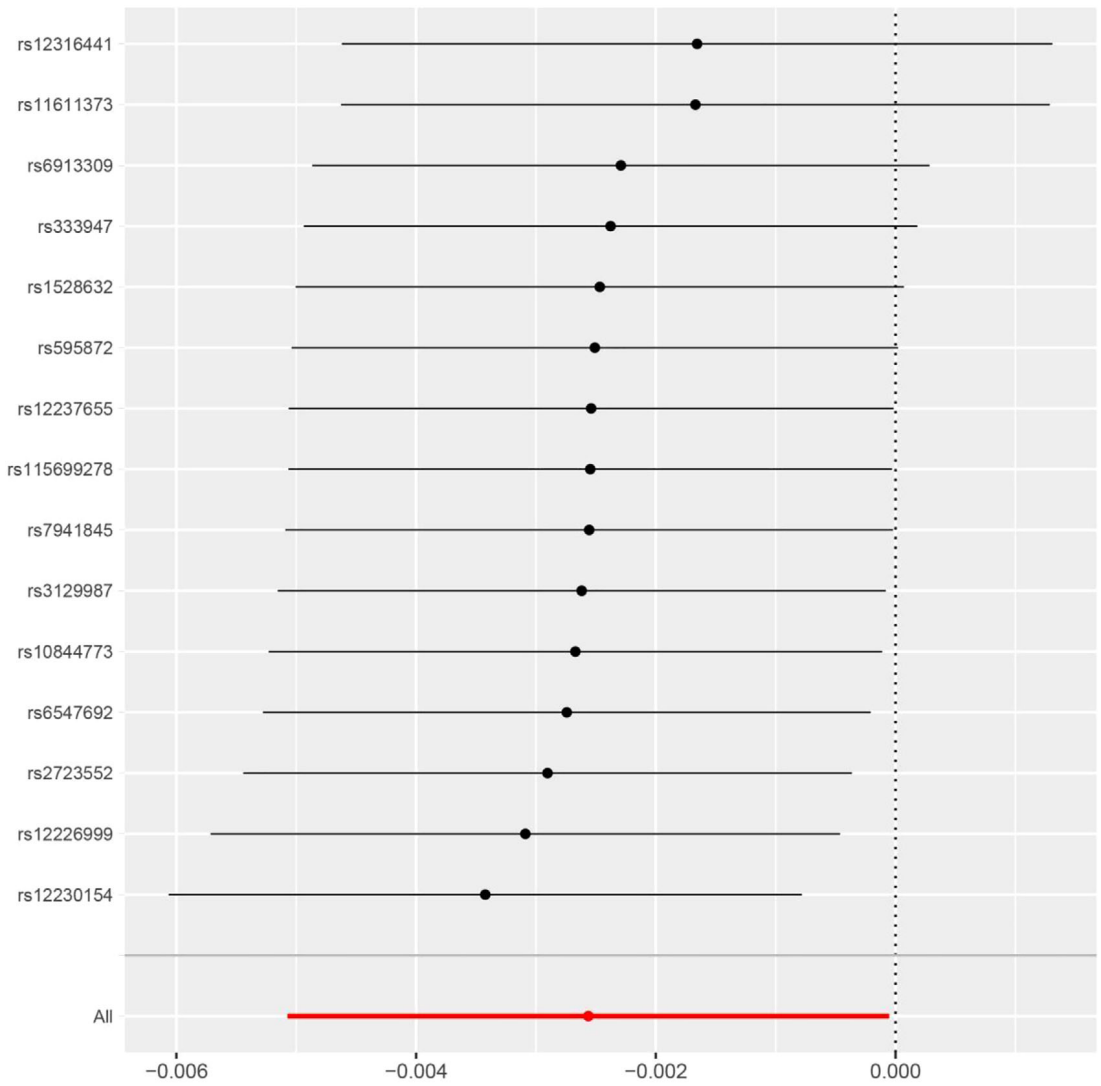
Leave‐one‐out of SNPs associated with LDH and their risk of IS. No single SNP is strongly driving the overall effect of LDH on IS in this leave‐one‐out sensitivity analysis.

From the results of the leave‐one method (the results are shown in Figure 3), no single SNP plays a decisive role in causal reasoning. Egger intercept was 0.00016, SE was 0.00012, and *p*‐values were .182. The resultant value for IVW was 11.1 (*p* = .6022) and the Q resultant value for MR egger was 13.09 (*p* = .5194). The *p*‐value was greater than .05, indicating that there was no horizontal pleiotropy in the resultant outcomes.

## 4 DISCUSSION

IS is a disorder of energy metabolism due to occlusion of blood supply arteries to the brain, resulting in reduced energy and oxygen supply. It is a disease that eventually leads to brain tissue necrosis in the brain and even death. With the gradual increase in the trend of population aging, the prevalence of IS is increasing year by year, but the role and causality of LDH in IS is unclear. Classical epidemiological studies have difficulty explaining the causal sequence of exposure factors and clinical outcomes due to the influence of confounding factors. In contrast, MR minimizes the bias of observational studies (Lawlor et al., 2008). The aim of this study was to investigate the relationship between LDH and IS through a two‐sample MR study.

In the present study, we used the MR study of GWAS to explore the relationship between LDH and the risk of developing IS. We then selected 15 SNPs significantly associated with LDH as instrumental variables. MR analysis was performed using the IVW method, weighted median estimation method, and MR‐egger method. We finally combined the data from the GWAS study of IS and concluded that there was an inverse relationship between LDH and increased risk of IS prevalence. Our results are contrary to previous observational studies which suggested LDH was positively associated with IS.

The most important etiology of IS is atherosclerosis. Atherosclerosis is closely associated with diseases such as hypertension and diabetes mellitus, and the endothelial damage caused by these diseases promoting atherosclerotic plaque formation. The results of past observational studies concluded that LDH levels were elevated after atherosclerosis and IS (Hazbar & Sahab, 2018; Wang et al., 2021), and the elevation of LDH is caused by endothelial damage. However, our conclusion suggests an inverse association between LDH and IS development. This opposite conclusion may be due to the bias of observational studies. Risk factors for IS, such as atherosclerosis, may be present in cases with high LDH. The risk of IS development within one year was significantly higher in patients with high LDH than in the normal LDH group. Therefore, observational studies have concluded that patients with high LDH are susceptible to IS. Such conclusions may be influenced by the factor of bias.

Recent studies have found that LDH plays an important role in maintaining vascular homeostasis (Parra‐Bonilla et al., 2013). Vascular endothelial cells are different from other cells in that their energy supply is provided mainly by glycolysis. The final step of glycolysis is the conversion of pyruvate to lactate catalyzed by LDH, which is also involved in maintaining a high glycolytic rate and is transported to the extracellular space via monocarboxylate transport proteins to prevent the accumulation of harmful substances in the cell. LDH silencing significantly reduces angiogenesis, suggesting that LDH is essential for maintaining vascular endothelial cells, vascular homeostasis and angiogenesis (Parra‐Bonilla et al., 2013).

Glycolysis is the main source of energy for endothelial cells (Ali et al., 2018), and elevated NADH/NAD+ ratios inhibit glycolysis (Chen et al., 2016). LDH converts pyruvate and NADH to lactate and NAD+, which increases glycolysis to supply energy to endothelial cells and maintain endothelial energy supply. However, the level of glycolysis is significantly increased in atherosclerosis after endothelial injury (Ali et al., 2018), causing inflammation that exacerbates endothelial cell damage. Higher LDH levels within normal levels are beneficial for maintaining vascular endothelial function. However, high LDH is detrimental after the onset of atherosclerosis and IS, which is consistent with classical epidemiological findings (Hazbar & Sahab, 2018). Thus, relatively high LDH levels within normal levels are protective on the vascular endothelium before the onset of IS and IS risk factors (i.e., hypertension, hyperlipidemia, and atherosclerosis, etc.). This is because LDH enhances the energy supply of endothelial cells to protect vascular homeostasis. Therefore, LDH is inversely associated with the risk of developing IS. LDH levels may be modulated by a variety of factors, such as ethnicity, hyperlipidemia, diabetes, and obesity (Ciofani et al., 2023). In the future, more observational studies in different ethnic groups are needed to validate the causal relationship between LDH and IS under the condition of controlling bias.

Inevitably, the present study has some limitations. First, the sample size included in the MR analysis was small. Therefore, the findings have yet to be confirmed by a prospective study with a larger sample. Second, due to limitations of the pooled data, detailed information at the individual level could not be obtained. Moreover, rs12316441 and rs11611373 were not significant in the sensitivity analysis. Therefore, rs12316441 and rs11611373 had a very large effect on the total data, which may make the results less reliable. This becomes a limitation of this study, demonstrating that the evidence for an inverse association between LDH and IS risk may be not strong enough, and more observational studies are needed to validate it in the future. In observational studies, the focus should be on the risk of developing IS with high and low LDH within the normal range. Our findings suggest that LDH may play an important role in the development of IS (especially in the early stages of the pathological process). The findings of this study may provide an opportunity to investigate the potential mechanisms underlying the effects of LDH on IS.

## 5 CONCLUSIONS

With Mendelian randomization, we have found that there was an inverse causal association between LDH and IS. However, further research is need to complement these findings, and the underlying mechanism of this causal relationship needs further study.

## AUTHOR CONTRIBUTIONS

XW initiated the study, participated in the study design, organized data extraction, and wrote the first draft of the paper. FXD contributed to article writing and data analysis. WX and FXD contributed equally to this study. JJL contributed to article writing. DZX and JHL initiated the study and contributed in supervising, writing and revising the paper. All authors contributed to the article and approved the submitted version.

## CONFLICT OF INTEREST STATEMENT

The authors declare that there is no conflict of interest regarding the publication of this article.

### PEER REVIEW

The peer review history for this article is available at https://publons.com/publon/10.1002/brb3.3352.

## Data Availability

The data for this study can be provided by the corresponding author (Jinhua Li: jinhua1@jlu.edu.cn).
